# Association between human leukocyte antigen-DQ2/DQ8 positivity and incident atrial fibrillation in patients with heart failure with reduced ejection fraction

**DOI:** 10.1590/1806-9282.20260404

**Published:** 2026-08-24

**Authors:** Uğur Küçük, Ercan Akşit

**Affiliations:** 1 Canakkale Onsekiz Mart University, Faculty of Medicine, Department of Cardiology – Çanakkale, Turkey.

**Keywords:** HLA-DQ2, HLA-DQ8, Atrial remodeling, Inflammation, Genetic susceptibility

## Abstract

**OBJECTIVE:**

Genetic susceptibility is increasingly recognized in cardiac arrhythmias. human leukocyte antigen-DQ2 and human leukocyte antigen-DQ8 alleles are established markers of immune-genetic dysregulation and chronic inflammation. This study investigated the association between human leukocyte antigen-DQ2/DQ8 positivity and incident atrial fibrillation in patients with heart failure with reduced ejection fraction.

**METHODS:**

This study was based on a prospectively followed cohort; the present retrospective analysis evaluated 50 heart failure with reduced ejection fraction patients (left ventricular ejection fraction <40%) and 50 age- and sex-matched controls. Participants were screened for human leukocyte antigen-DQ2/DQ8 alleles. All subjects were in sinus rhythm at baseline and followed for 24 months for the primary endpoint of incident atrial fibrillation.

**RESULTS:**

Incident atrial fibrillation occurred in 16% (n=8) of the heart failure with reduced ejection fraction group and 2% (n=1) of the control group. Among heart failure with reduced ejection fraction patients, human leukocyte antigen-positive status (positivity for DQ2 and/or DQ8) was found in 20 patients. Interestingly, all eight incident atrial fibrillation cases in the heart failure with reduced ejection fraction group occurred within the human leukocyte antigen-positive subgroup (40 vs. 0% in human leukocyte antigen-negative heart failure with reduced ejection fraction, p=0.031). human leukocyte antigen-positive heart failure with reduced ejection fraction patients exhibited a significantly higher atrial fibrillation risk compared to human leukocyte antigen-positive controls (p=0.0069), suggesting that structural heart disease and genetic predisposition synergistically increase arrhythmic risk.

**CONCLUSION:**

Human leukocyte antigen-DQ2/DQ8 positivity is significantly associated with incident atrial fibrillation in heart failure with reduced ejection fraction patients. These findings suggest human leukocyte antigen-related immune-genetic susceptibility contributes to atrial electrical vulnerability, positioning human leukocyte antigen typing as a potential novel biomarker for arrhythmic risk stratification in heart failure.

## INTRODUCTION

Heart failure (HF) is frequently complicated by atrial fibrillation (AF), which is associated with adverse clinical outcomes, increased hospitalization rates, and impaired quality of life^1,2^. While structural remodeling and neurohormonal activation are recognized contributors to arrhythmogenesis, genetic and immunologic susceptibility factors remain insufficiently explored.

Human leukocyte antigen (HLA) polymorphisms play a critical role in immune-mediated cardiovascular remodeling and inflammatory signaling^3^. Previous studies have suggested associations between specific HLA haplotypes and myocardial disease susceptibility, conduction abnormalities, and arrhythmogenic risk^4,5^. Therefore, HLA-DQ2/DQ8, well-known markers of systemic inflammatory responses and autoimmune dysregulation, may modulate the threshold for atrial electrical instability. However, the relationship between HLA-DQ2/DQ8 positivity and AF development in HF populations has not been systematically investigated.

The present study aimed to evaluate whether HLA-DQ2/DQ8 positivity is associated with incident AF during longitudinal analysis of clinical outcomes in patients with heart failure with reduced ejection fraction (HFrEF). By focusing on incident AF rather than prevalent arrhythmia, this study seeks to identify a genetically susceptible arrhythmia phenotype in HF.

## METHODS

### Study design

This study represents a distinct, long-term secondary analysis of a prospectively followed single-center clinical cohort. The baseline recruitment methodology, initial patient characteristics, and the cross-sectional distribution of HLA-DQ2/DQ8 haplotypes have been previously described in detail in our prior publication^6^. While our prior investigation was strictly a cross-sectional analysis focused on comparing baseline genetic prevalence, the present retrospective analysis was specifically designed to evaluate a completely novel, longitudinal clinical endpoint.

The present analysis evaluated 50 consecutive patients hospitalized with HFrEF [left ventricular ejection fraction (LVEF) <40%] and 50 age- and sex-matched control subjects from the original cohort. Controls comprised apparently healthy individuals with an LVEF ≥40% and no overt or subclinical cardiovascular diseases. The inclusion of the age- and sex-matched healthy control group served a dual purpose: first, to establish the baseline demographic prevalence of the HLA-DQ2/DQ8 alleles, and second, to demonstrate the synergistic arrhythmogenic effect of genetic susceptibility when combined with the structural substrate of HFrEF. All participants were in sinus rhythm at baseline.

**Figure 1:**
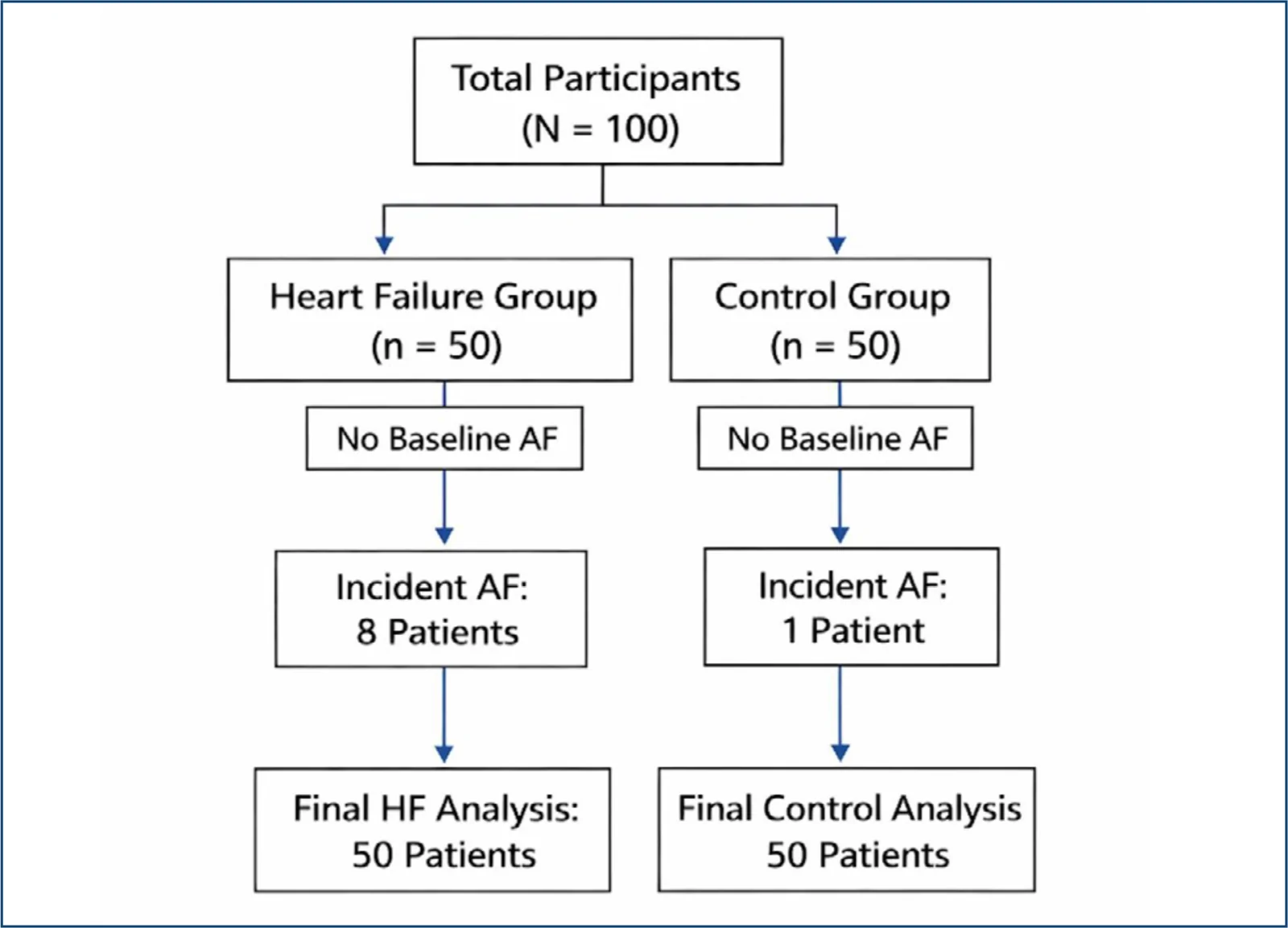
Flowchart of the study population.

Since HLA-DQ2/DQ8 screening isn't routine in cardiology, our cohort underwent testing only for specific immune or clinical indications (e.g., unexplained chronic anemia, gastrointestinal symptoms, or suspicion of concurrent autoimmune disorders). HFrEF patients without these test results were excluded from this retrospective analysis. We applied identical immunogenetic criteria to both HFrEF and control groups, classifying patients with DQ2 or DQ8 alleles as HLA-positive. The patient selection process is summarized in Figure 1.

During the 24-month follow-up (3–6-month intervals), routine clinical and 12-lead electrocardiogram (ECG) assessments occurred. Holter monitoring and device interrogations were reserved for clinical indications.

HF therapy was optimized during follow-up, with angiotensin receptor–neprilysin inhibitors, angiotensin-converting enzyme inhibitors/angiotensin receptor blockers (ACEI/ARB), beta-blockers, sodium–glucose cotransporter 2 inhibitors, and diuretics titrated to guideline-recommended target doses^1^.

Exclusion criteria for the patient group were the following: (i) age <18 years or unwillingness to participate; (ii) acute or chronic liver disease, severe renal failure defined as estimated glomerular filtration rate (eGFR) of <30 mL/min/1.73 m²; (iii) hepatitis B or C infection, inflammatory and hematological diseases, active thyroid disease, cancer, autoimmune thyroiditis, collagen tissue diseases; (iv) heart valve disease (moderate or severe), genetic cardiomyopathy (hypertrophic cardiomyopathy, left ventricular noncompaction, restrictive cardiomyopathy), structural heart disease, toxin-induced cardiomyopathy, or peripartum cardiomyopathy; (v) suspected pregnancy; and (vi) celiac disease.

### Clinical, laboratory, and echocardiographic data collection

Peripheral venous blood was collected in ethylenediaminetetraacetic acid (EDTA) tubes. After DNA extraction using the QIAamp DNA Blood Mini Kit, real-time polymerase chain reaction (PCR) was performed with the geneMAP Celiac detection kit and Qiagen Rotor-Gene Q protocol. After the completion of the run, the data were analyzed using melting curve analysis with Rotor-Gene Q series software (QIAGEN) to detect HLA haplotypes. Since routine HLA-DQ2/DQ8 screening is uncommon in cardiology, testing was limited to patients with specific clinical indications (e.g., anemia, gastrointestinal symptoms, and autoimmune suspicion) or immune-cardiology evaluations. Consequently, systematically followed HFrEF patients lacking these results were excluded from this retrospective analysis. Baseline demographics, clinical data, comorbidities, medications, laboratory values, and echocardiograms were retrieved from institutional electronic medical records. Collected clinical variables included age, sex, HF etiology (ischemic vs. non-ischemic), blood pressure measurements, renal function parameters (serum creatinine and eGFR), serum sodium, hemoglobin, and N-terminal pro-B-type natriuretic peptide (NT-proBNP) levels. Transthoracic echocardiographic examinations were performed according to current guideline recommendations. LVEF was calculated using the modified Simpson method^7^.

### Follow-up and outcome definition

All patients were followed for up to 24 months after the index hospitalization using outpatient clinic records and hospital electronic databases. Throughout the 24-month follow-up period, patients were systematically evaluated during routine outpatient HF clinic visits scheduled every 3–6 months. At each visit, clinical status was assessed, and a standard 12-lead ECG was obtained. Additionally, 24-h Holter monitoring was performed when clinically indicated by symptoms such as palpitations or unexplained clinical deterioration.

### Definition of incident atrial fibrillation

Incident AF was defined as the first documented episode of AF occurring during the follow-up period in patients without a prior history of AF at baseline. AF diagnosis was confirmed by at least one of the following: a standard 12-lead ECG, an episode lasting ≥30 s on Holter monitoring or inpatient telemetry recordings, or device-detected atrial high-rate episodes in patients with implantable cardiac devices, subsequently documented by a cardiologist in the electronic medical record. Routine outpatient ECG recordings were obtained during scheduled follow-up visits. Holter monitoring and device interrogations were performed when clinically indicated. When available, the date of the first documented AF episode was recorded as the event time.

The local Ethics Committee approved this study (approval no: 2026-02/02-18). The study followed the ethical guidelines outlined in the Declaration of Helsinki. Informed consent was obtained both verbally and in writing.

### Statistical analysis

Continuous variables were expressed as mean±SD or median (IQR). Categorical variables were expressed as counts and percentages. Group comparisons were performed using an independent sample t-test, Mann-Whitney U test, chi-square test, or Fisher's exact test as appropriate. Kaplan-Meier survival analysis with log-rank testing was used for AF-free survival. Haldane-Anscombe correction was applied to calculate odds ratios when zero-event cells were present. A p-value <0.05 was considered statistically significant.

## RESULTS

A total of 100 participants were included in the final analysis, comprising 50 patients with HFrEF and 50 control subjects. Baseline clinical and echocardiographic characteristics are summarized in Table 1. There were no statistically significant differences between groups with respect to age, heart rate, systolic or diastolic blood pressure, or sex distribution. The prevalence of HLA-DQ2/DQ8 positivity was comparable between the HF and control groups (40.0 vs. 46.0%, p=0.686) (Table 1). Body mass index was similar across the evaluated groups (Table 1). Upon review of the clinical records, none of the participants in either the HFrEF or the control group had a documented history of obstructive sleep apnea (OSAS). During follow-up, incident AF occurred significantly more frequently in the HF group than in controls (16.0 vs. 2.0%, p=0.031) (Table 1).

**Table 1:** Baseline clinical characteristics of the study population.

Variable		Heart failure group (n=50)	Control group (n=50)	p-value
Age (years)		62.2±15.0	59.7±13.6	0.375
Male sex, n (%)		36 (72.0%)	28 (56.0%)	0.145
BMI (kg/m^2^)		28.10±1.32	27.68±1.87	0.198
Echocardiographic data				
	LVEF (%)	28.3±8.0	57.7±5.5	<0.001
	LVEDV (mL)	135.12±8.15	88.37±3.26	<0.001
	LVESV (mL)	91.68±3.18	61.3±4.1	<0.001
	LA (mm)	38.2±4.1	33.2±2.8	<0.001
	LAVi (mL/m^2^)	38.4±2.3	29.8±2.7	<0.001
	Heart rate (bpm)	83.4±12.2	81.2±11.8	0.362
	Systolic BP (mmHg)	119.4±20.5	125.6±12.9	0.076
	Diastolic BP (mmHg)	72.4±8.2	72.8±8.3	0.837
	HLA-DQ2/DQ8 positivity, n (%)	20 (40.0%)	23 (46.0%)	0.686
	Incident atrial fibrillation, n (%)	8 (16.0%)	1 (2.0%)	0.031

BMI: body mass index; LVEF: left ventricular ejection fraction; LVEDV: left ventricular end-diastolic volume; LVESV: left ventricular end-systolic volume; LA: left atrium; LAVi: left atrial volume index; BP: blood pressure; HLA: human leukocyte antigen.

When the HF cohort was stratified according to HLA-DQ2/DQ8 status, a striking difference in AF incidence was observed. Among HLA-positive HF patients (n=20), AF developed in 8 patients (40.0%), whereas no AF events occurred in HLA-negative HF patients (n=30) (p=0.0002) (Table 2). In secondary analyses, AF incidence remained significantly higher in the total HF cohort compared with the control group (16.0 vs. 2.0%, p=0.0309) (Table 2). In a subgroup analysis of HLA-positive HFrEF patients (n=20), no significant differences were observed between those who developed incident AF (n=8) and those who remained in sinus rhythm (n=12) regarding age, left atrium diameter, and left atrial volume index (all p>0.05). This suggests that HLA positivity may predispose to AF independently of traditional structural and demographic risk factors within the HF population.

**Table 2:** Incident atrial fibrillation according to human leukocyte antigen-DQ2/DQ8 positivity.

Group	AF (+) n (%)	AF (-) n (%)	Total (n)	p-value*
HF+HLA-DQ2/DQ8 positive	8 (40.0%)	12 (60.0%)	20	0.0002
HF+HLA-DQ2/DQ8 negative	0 (0.0%)	30 (100.0%)	30	Reference
Total HF cohort	8 (16.0%)	42 (84.0%)	50	-
Control group	1 (2.0%)	49 (98.0%)	50	0.0309

* Fisher's exact test.

Primary comparison: HF HLA-positive vs. HF HLA-negative. Secondary comparison: Total HF vs. Control (p=0.0309). For HLA-positive HF vs. HLA-positive Control: p=0.0069. Odds ratio for the primary comparison is undefined due to a zero-event cell; Haldane-Anscombe corrected OR 41.48 (95%CI 2.22–774.64). AF: atrial fibrillation; HF: heart failure; HLA: human leukocyte antigen.

Due to the absence of AF events in the HLA-negative HF subgroup, the crude odds ratio for the primary comparison was not directly estimable. After applying the Haldane-Anscombe correction, HLA positivity in HF patients was associated with a markedly increased risk of incident AF (corrected OR 41.48, 95%CI 2.22–774.64), highlighting the strong magnitude of association despite the limited event number.

## DISCUSSION

This study provides novel evidence that HLA-DQ2/DQ8 positivity is strongly associated with incident AF in patients with HFrEF. Notably, AF developed exclusively among HLA-positive patients, suggesting that these alleles may identify a genetically susceptible arrhythmogenic substrate that becomes clinically evident under the structural, neurohormonal, and inflammatory stress of HF. This finding supports the concept of atrial cardiomyopathy, whereby genetic predisposition interacts with acquired pathological remodeling to promote electrical instability^8,9^.

Several mechanisms may explain this association. HLA-DQ2 and HLA-DQ8 are linked to enhanced antigen presentation and chronic immune activation^10^. Persistent low-grade inflammation may contribute to atrial fibroblast activation, extracellular matrix remodeling, and interstitial fibrosis, all of which are recognized substrates for AF^11–13^. Because HFrEF itself is characterized by systemic inflammation, oxidative stress, and neurohormonal activation, the coexistence of HLA-mediated immune susceptibility may further amplify atrial structural and electrophysiological remodeling, potentially explaining why AF occurred only in HLA-positive individuals in our cohort.

Our findings are consistent with previous evidence linking inflammation and immune-mediated disorders to AF development^14–16^. However, data regarding specific HLA haplotypes and arrhythmic outcomes in HFrEF remain limited. Therefore, this study extends current knowledge by identifying HLA-DQ2/DQ8 positivity as a potential immunogenetic marker associated with incident AF in a high-risk HF population. Although the confidence intervals were wide because of the limited number of events, the observed association remained consistent across analyses^17^.

From a clinical perspective, current AF risk stratification in HF relies mainly on clinical and echocardiographic parameters^18^. Incorporating immunogenetic markers may improve the identification of patients with increased arrhythmic vulnerability who could benefit from closer rhythm surveillance or prolonged ambulatory monitoring. Interestingly, among HLA-positive patients, traditional AF predictors such as age and left atrial size did not differ significantly between those who developed AF and those who did not, suggesting that HLA-DQ2/DQ8 positivity may identify risk beyond conventional structural markers.

Our findings highlight an intriguing demographic interplay: while HFrEF and AF traditionally affect older males, HLA-DQ2/DQ8-linked conditions typically affect younger females^19,20^. These alleles may accelerate arrhythmogenic remodeling, potentially shifting AF vulnerability to a younger demographic or altering traditional gender ratios. Future large-scale studies are required to confirm this.

Several study limitations should be noted. The single-center, small-sample design yields low statistical power, making the findings strictly exploratory. Requiring prior HLA testing introduces selection bias, while standard rhythm monitoring may underestimate short or asymptomatic AF episodes^17^. Furthermore, unmeasured confounders like undiagnosed OSAS, dynamic inflammation, and autonomic imbalance cannot be definitively excluded. Finally, the single-time HLA assessment misses potential dynamic immune changes over time.

Despite these limitations, strengths include a prospective design, strict baseline AF exclusion, objective genotyping, and consistent risk estimates. Clinically, we advise against universal HLA screening in HFrEF. However, for HFrEF patients with known HLA-DQ2/DQ8 positivity or related autoimmune conditions, this genetic trait is a major risk modifier. These high-risk patients warrant intensive rhythm surveillance, potentially using wearable monitors for early AF detection and timely anticoagulation.

Future multicenter studies with larger cohorts and continuous rhythm monitoring are warranted to validate these findings and to explore whether HLA-guided risk stratification can improve arrhythmia prevention strategies in HF populations.

## CONCLUSION

To our knowledge, this is the first study linking HLA-DQ2/DQ8 positivity to incident AF in HFrEF patients over 24 months. While not proving causality, these hypothesis-generating findings support the concept of immune-mediated atrial myopathy in HF. Identifying this genetic susceptibility highlights a high-risk phenotype needing vigilant rhythm monitoring.

## Data Availability

The datasets generated and/or analyzed during the current study are available from the corresponding author upon reasonable request.
